# Application of Dispersive Liquid–Liquid Aerosol Phase Extraction to the Analysis of Total and Individual Phenolic Compounds in Fried Extra Virgin Olive Oils

**DOI:** 10.1021/acs.jafc.3c02634

**Published:** 2023-07-03

**Authors:** Raquel Sánchez, Ana Beltrán Sanahuja, María Soledad Prats Moya, José-Luis Todolí

**Affiliations:** Department of Analytical Chemistry, Nutrition and Food Sciences, P.O. Box 99, 03080 Alicante, Spain

**Keywords:** extra virgin olive oil, polyphenols, degradation rate, frying, dispersive liquid−liquid aerosol phase extraction

## Abstract

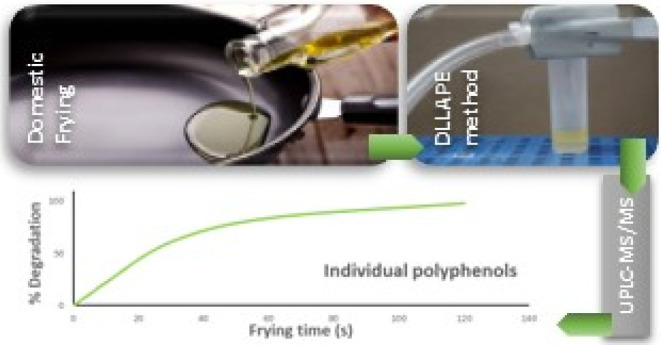

Seventeen extra virgin olive oil samples from Valencian Community (Spain) were submitted to a domestic-frying process (180 °C) during different degradation times (5, 10, 30, 60, 120 min). A dispersive liquid–liquid aerosol phase extraction by using a methanol/water (50:50) extracting solution was used to isolate the polyphenol fraction. Total phenolic content (TPC) was determined, whereas the determination of seven individual target polyphenolic compounds (hydroxytyrosol, tyrosol, oleuropein, vanillic acid, *p*-coumaric acid, ferulic acid, and vanillin) was carried out by using ultrahigh-performance liquid chromatography coupled to a tandem mass spectrometer. Statistically significant differences in the TPC values were found for Blanqueta and Manzanilla samples from different harvesting years. The domestic-frying process impacted the TPC and the individual phenolic compounds content. Thermal treatment for 2 h gave rise to a 94% decrease in the TPC. A first-order kinetic model was suitable to accurately describe the degradation of the individual phenolic compounds.

## 1 Introduction

Olive growing in the Mediterranean region is a longstanding tradition partly because of its ability to tolerate dry edaphoclimatic conditions.^1^ Olive oil is appreciated worldwide, and it is produced and consumed mainly in the south of Europe. According to Food and Agriculture Organization Corporate Statistical Database(FAOSTAT), the world’s virgin olive oil production in 2020 was around 3.1 million tons. By continents, Europe contributed nearly 63% to the world production, and by countries, Spain (1.13 million tons) enjoys a leadership position followed by Italy and Greece (0.34 and 0.29 million tons, respectively).^2^

Around the world, there is a wide range of olive cultivars; some of the most popular ones are grown in Spain and Italy.^1^ Among all olive cultivars cultivated in Spain, the most popular ones in decreasing growing extension are the following: Picual, Cornicabra, Hojiblanca, Lechin, Arbequina, Manzanilla de Sevilla, Morisca, Empeltre, Manzanilla Cacereña, Picudo, Farga, Lechin de Granada, Verdial de Huevar, Gordal Sevillana, Verdial de Badajoz, Morrut, Sevillenca, Castellana, Verdial de Velez Rubio, Aloreña, Blanqueta, Villalonga, Changlot Real, and Alfafara.^1^

Virgin olive oil is the juice obtained directly from olives, extracted exclusively by physical processes, without the addition of solvents for optimizing the extraction yield.^3^ Since the refining process is not applied, this will ensure that some important antioxidants are directly transferred from the olive fruit to the oil, thus contributing to its high antioxidant capacity. The most relevant antioxidant compounds present in olive oils are vitamin E, carotenes, and polyphenols.^4^ More specifically, virgin olive oils are rich in a wide variety of polyphenolic compounds, from complex molecules like flavonoids, hydroxy-isochromans, secoiridoids, and lignans to more simple molecules such as simple phenols and phenolic acids.^5^ Phenolic acids are found in low quantities in olive oil but are known to have strong antioxidant properties. According to the phenol explorer database,^6^ the phenolic acids most usually found in virgin olive oil are *p*-coumaric (0.1–3.6 mg kg^–1^), caffeic acid (0–1.5 mg kg^–1^), vanillic acid (0.2–1.4 mg kg^–1^), syringic acid (0–0.6 mg kg^–1^), and ferulic acid (0–0.5 mg kg^–1^) among others. Additionally, tyrosol (TYR) and hydroxytyrosol (HYR) are the simple phenols in virgin olive oils present at higher quantities ranging from 0.1 to 35.6 and 0.7 to 34.7 mg kg^–1^, respectively.^7^ Besides, the most abundant polyphenols are the secoiridoid group, the main ones being oleuropein-aglycone monoi-aldehyde (3,4-DHPEA-EA), oleuropein-aglycone di-aldehyde (3,4-DHPEA-DEA), and ligstroside-aglycone di-aldehyde (*p*-HPEA-EDA), among others. Depending on the olive oil, these mentioned groups of compounds can represent more than 50 mg kg^–1^ altogether, 3,4-DHPEA-EDA being the most abundant in olive oils.^6,8^

For the frying process, the importance of polyphenols lies in the influence they exert on the oxidation resistance of vegetable oils.^9^ In this line, previous studies have shown a significant reduction in the amount of antioxidants in olive oil after heating.^9−11^ Temperature reached in the cooking process seems to be the most relevant variable in the degradation of virgin olive oil polyphenols. However, the presence of foods in the cooking process may mask the actual impact of temperature on the degradation of polyphenols from extra virgin olive oil (EVOO). Thus, for instance, when the oil was used in sautéing at 120 °C, the content of some specific polyphenolic compounds changed, although the total polyphenol content remained virtually unaltered. This fact was explained by the transformation of the most complex secoiridoids, such as oleuropein, into simpler compounds. Besides, when sautéing was done at higher temperatures like 170 °C, a clear degradation of all individual polyphenols was observed also leading to a decrease in total polyphenols.^4^ This behavior was also observed by Casal et al.^9^ after 6 h of using olive oil in a frying process at 170 °C. Under these conditions, all tocopherols and polyphenols disappeared, and only 50% of the original carotenes persisted. Following this line, Criado-Navarro et al.^12^ measured the concentration of major phenolic compounds in samples of monocultivar EVOOs (Arbequina, Cornicabra, Hojiblanca, and Picual) subjected to frying for 90 min at 180 °C. A significant decrease in the phenolic content was reported, and changes in the phenolic profile were detected by the conversion of open forms of oleuropein and ligstroside aglycones to oleacein and oleocanthal in the first frying cycles. In relation to longer frying times, previous studies have confirmed a similar decrease in polyphenol content for 1 or 5 h, indicating that longer times did not affect the phenolic compounds loss.^13^

Therefore, in the presence of food, several factors, such as the partial evaporation of food moisture (that can increase the concentration of phenolic compounds in the food matrix), the migration toward media with different polarity, and changes in the microstructure of the food sample among others, are relevant in the evaluation of the effects of olive oil cooking on phenolic compounds.^14^ Given the large number of factors involved in the process, the situation becomes confusing if the influencing factors are not considered separately.^12,15^

To perform polyphenols determination, a first isolation step is required. In fact, their efficient extraction is one of the most critical steps of the analytical method. The most used extraction process is the liquid–liquid extraction employing different organic solvents such as mixtures of methanol or ethanol with water.^16−19^ Solid-phase extraction has also been employed to isolate the phenolic fractions from olive oils.^18^ Recently, the fast dispersive liquid–liquid aerosol phase extraction (DLLAPE) method was applied by our research group to the analysis of EVOO samples.^17^ The aerosol generated from the extractant solution allowed a bigger exchange surface area with the sample. As a result, the polyphenol extraction yield was similar to that for the classical liquid–liquid extraction method, the analysis time being shorter and the required amount of organic solvents being much lower for the DLLAPE procedure.

There are a limited number of studies considering the impact of temperature of oil heating (in the absence of foods) on individual polyphenols content.^12,13^ The present study aims at applying the DLLAPE method to the analysis of phenolic compounds present in seventeen different EVOO samples obtained from the Valencian Community (Spain) and to determine changes in the EVOO polyphenolic profile during a domestic frying process, making special emphasis on minor phenolic compounds since they are those for which a more important lack of information has been detected. The phenolic profile was measured by using ultrahigh-performance liquid chromatography coupled to a tandem quadrupole mass spectrometer (UHPLC-MS/MS), providing information on how the phenolic profile changed and how individual polyphenols degraded at different rates.

## 2 Materials and Methods

### 2.1 Reagents and EVOO Samples

Ultrapure water supplied by a three-step ion-exchange system, Milli-Q, fed by reverse osmosis, Elix 3, both from Millipore (El Paso, TX), was used to prepare extracting solutions. Methanol, analytical grade (Panreac, Barcelona, Spain), was also selected for the extracting solutions. Standards of gallic acid (Merck, Darmstadt, Germany) were prepared by proper dilution. Analytical-grade sodium carbonate (Panreac, Barcelona Spain) was used to basify the extracted solutions before the addition of Folin-Ciocalteu reagent (Merck, Darmstadt, Germany) to determine the total polyphenol content. HYR, TYR, oleuropein, *p*-coumaric acid, ferulic acid, vanillic acid, and vanillin were purchased from Sigma-Aldrich (St. Louis, MO, USA). For individual polyphenolic determination, acetic acid, acetonitrile, formic acid, and isopropanol were purchased from Fluka (St. Louis, MO, USA).

Table 1 summarizes the information about the codification of the EVOO samples together with their varietal composition and aging of harvesting. All EVOO samples were from Valencian Community (Spain). Seventeen EVOO samples were studied containing mainly Alfafara, Blanqueta, Changlot Real, Genovesa, Manzanilla, Morrut, Picual, and Villalonga olive cultivars. Many of the EVOO samples were monovarietal, and only three samples were obtained through blending some of the cultivars. Samples were obtained directly from local producers following a two-phases production methodology. According to the information provided by the manufacturers, the fruits were first washed with water and then milled and pressed, thus giving rise to two phases. Then a centrifugation step in a decanter was applied to separate the oil from the solid phase. Finally, washing water was added, and the oil–water mixture was centrifuged, again, thus providing the olive oil. All processes were performed at room temperature (i.e., cold production). Samples were kept in their bottles in the dark and at room temperature until their analysis.

**Table 1:** Codification of the EVOO Samples, Varietal Composition, and Aging

oil code	varietal composition	aging
A1	Alfafara	2019
A2	Alfafara	2019
B1	Blanqueta	2019
B2	Blanqueta	2020
B3	Blanqueta^a^	2021
B4	Blanqueta	2021
CR1	Changlot Real	2019
G1	Genovesa	2021
M1	Manzanilla	2018
M2	Manzanilla	2021
Mo1	Morrut	2019
P1	Picual	2021
P2	Picual	2021
V1	Villalonga	2019
V2	Villalonga	2021
GA1	Grossal, Arbequina^a^	2019
C1	Manzanilla^b^, Genovesa, Alfafara, Blanqueta, Cuquello, Changlot Real	2019

a Organic production.

b Dominant variety.

### 2.2 EVOO Thermal Degradation Studies

Three subsamples of 100 mL of each EVOO were placed in a stainless-steel pan (15 cm diameter) and submitted to a domestic-frying process at 180 °C without controlling the amount of light and oxygen. Different thermal degradation times (5, 10, 30, 60, or 120 min) were evaluated. The pan temperature was maintained during the whole cooking process.

### 2.3 Aerosol Phase Extraction (DLLAPE) Procedure

For sample preparation, polyphenols were extracted by following an aerosol phase extraction (DLLAPE) procedure.^17,20,21^ Under these conditions, polyphenol extraction occurred at the interface of each generated droplet of the extracting solution. 1 g of EVOO sample was poured into a 5 mL polypropylene extraction vial, and then 1 mL of hexane was added. The optimized methanol/water extracting solution (Section 3.1) was delivered to a glass pneumatic concentric nebulizer (TR-30-A2, Meinhard Glass Products, Santa Ana, CA). The optimum nebulization conditions were taken from Mirón et al.^17^ (Table S13). The nebulizer gas and liquid flow rates were adjusted at 0.3 L min^–1^ and 0.9 mL min^–1^, respectively, by using a mass flow controller (58505, Brooks Instruments, Hatfield, PA, USA) and a peristaltic pump (Perimax, Spetec, Erding, Germany). The extracting solution was aspirated and nebulized over the sample for 90 s. Then, after a few seconds, the two liquid phases separated, and the methanolic solution was taken with a pipette.

For total polyphenol content determination, the extracts were diluted with 50:50% (w/w) methanol/water prior to their analysis. However, the extract was directly analyzed for individual polyphenol content determination. Once the aerosol was generated at the nebulizer nozzle, solvent evaporation from the droplet surface begun. Consequently, a fraction of the extracting solution did not enter in contact with the sample. Approximately 30% of the 50:50% (w/w) methanol/water aerosol evaporated before reaching the sample surface. These losses were compensated for by weighing the tubes with their contents before and after the extraction step.^17^

### 2.4 Total Polyphenol Content Determination—Folin-Ciocalteu Method

A volume of 0.5 mL of the extract was added to 2 mL of the 50:50% (w/w) methanol/water extracting solution. To basify the solution, 0.2 mL of sodium carbonate 2.5% was added followed by the addition of 0.1 mL of the Folin-Ciocalteu reagent. Then, the mixture was kept in the dark for 30 min at room temperature. The absorbance was measured at 760 nm with a Thermo Fisher Scientific spectrophotometer (Orion AquaMate 7000 Vis, Valencia, Spain).

### 2.5 HPLC-DAD Analysis

In the first step, the composition of the extracting solution was optimized. And the methanolic extracts were analyzed by HPLC-diode-array detection (HPLC-DAD). The chromatographic analysis was performed using an Agilent 1260 (Santa Clara, CA, USA) series instrument, equipped with an autosampler, a binary solvent pump, and a diode–array detector (DAD). The separation was achieved on a Luna Omega reverse phase (150 × 4.6 mm, 5 μm) analytical column from Phenomenex (Chesire, UK). The mobile phase consisted of a mixture of water with 0.1% formic acid (A) and methanol/iPrOH 90:10 v/v with 0.1% formic acid (B) working in the gradient mode at a flow rate of 1 mL min^–1^. The solvent gradient applied was as follows: 0 min, 20% B; 0–30 min, 60% B; 30–45 min 95% B; 45–52 min 95% B; then the column was reconditioned during 5 min from 95% B to 20% B. The column temperature was set at 35 °C. The sample injection volume was 10 μL. HPLC-DAD was performed monitoring the absorbance at a 280 nm wavelength.

### 2.6 UHPLC-MS/MS Analysis

The analysis of individual polyphenols in the EVOO samples was carried out by UHPLC-MS/MS using an Agilent 1290 Infinity UHPLC System coupled to an Agilent 6490 triple quadrupole mass spectrometer (Santa Clara, CA, USA) with an Agilent Jet Stream ion source in negative ionization (NI) mode. Separation of analytes was performed on an Agilent Poroshell 120 EC-C18 column (Agilent Technologies, Santa Clara, CA 95051-7201, USA), 3 × 100 mm, 2.7 μm, which was maintained at 25 °C during the analysis. In optimized conditions, the mobile phase consisted of solvent A (0.01% acetic acid in water) and solvent B (0.01% acetic acid in acetonitrile) using the following gradient: 1 min, 53% B; 2.5 min, 53.5% B; 5 min, 54.2% B; 6 min, 95% B; 6.2 min, 20% B; at a constant flow rate of 0.2 mL min^–1^. For all samples, the injection volume was 2 μL.

The multiple reaction monitoring (MRM) analysis mode was used to monitor the transitions from precursor ions to dominant product ions. The optimized source parameters were as follows: gas curtain temperature 275 °C, gas flow 11 L min^–1^, cell acceleration voltage 4 V, nebulizer pressure 45 psi, capillary voltage 4000 V; fragmentor voltage 380 V; resolution first and second quadrupole 0.7 (unit); negative polarity and dwell time 10 ms. Several specific transitions were used to determine each compound, and for each transition, the collision energy applied was optimized to detect the greatest possible intensity. The specific MRM transitions used for quantification of each analyte and the collision energy are summarized in Table S1.

A MassHunter Workstation (version B.07.01) was used for data acquisition. MassHunter Qualitative Analysis (version B.07.00) and Quantitative Analysis Software (version B.07.00) were used for data processing. The most abundant MRM transitions were selected for each analyte as a quantifier and the other transitions as qualifier ions.

To evaluate the analytical features of the proposed method, calibration curves of the targeted minor compounds were constructed in the 0.1–115 μg kg^–1^ range at eight concentration levels. For HYR and TYR, the calibration curves were carried out in the 0.005–5 mg kg^–1^ concentration range. For the major compounds oleacin, oleocanthal, luteolin, oleuropein aglycone, ligstroside, and apigenin, the quantification was carried out by using oleuropein as standard.

Limits of detection (LOD) and quantification (LOQ) were estimated by applying the 3s_b_ and 10s_b_ criteria, respectively, where s_b_ is the standard deviation for 6 replicates of the least concentrated standard (0.1 μg kg^–1^). Five sub-samples of a quality control sample (25 μg kg^–1^) were measured on three different days, using five different calibration curves. One-way ANOVA was used to estimate the repeatability and intermediate precision as within-group and between-group standard deviations, respectively.

### 2.7 Evaluation of the Effect of Thermal Treatment on the Content of Polyphenols in EVOO Samples

An objective of the present work was the evaluation of the effect of home-frying treatment on the content of polyphenols in EVOO samples. With this purpose, the percentage of degradation was calculated by using the following equation
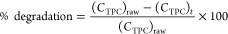
1where (*C*_TPC_)_raw_ is the total phenolic content, expressed as mg gallic acid equivalent kg^–1^ oil, for the raw EVOO; and (*C*_TPC_)_*t*_ is the total phenolic content after the thermal treatment.

Food thermal degradation could be described by using kinetic models, where changes of the phenolic content could be described by mathematical models containing kinetic parameters.^22^ Mostly, first-order kinetics is used to describe food thermal degradation
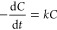
2where *C* is phenolic compound concentration, *t* is the time, and *k* is the reaction constant. Considering that *C*_0_ is the initial phenolic compound concentration and integrating eq 2, the following equations are obtained
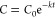
3
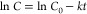
4

### 2.8 Statistical Analysis

SPSS (SPSS 28.0; Inc, Chicago, USA) statistical program was used to analyze the results. A one-way analysis of variance (ANOVA) was applied to compare the mean values of each compound and olive cultivar. Additionally, a pair-wise Tukey-b post hoc test at 0.05 significance level was employed to detect the values which were significantly different.

## 3 Results and Discussion

### 3.1 Aerosol Phase Extraction (DLLAPE) Optimization

Mirón et al.^17^ demonstrated the suitability of the aerosol phase extraction procedure (DLLAPE) for the determination of the total polyphenol content. As the extraction takes place in the interface between the sample and the surface of each individual droplet of the aerosol, a great effort was made to optimize the variables affecting the characteristics of the aerosols generated by the pneumatic nebulizer (i.e., liquid and gas flow rates) as well as those having an impact on the partition equilibrium (i.e., the mass and composition of extracting solution, nebulizer tip-sample distance, and extraction time).

The partition of polyphenols between the phases could be directly impacted by the composition of the extracting solution. The efficiency of extraction relies on the individual characteristics of each polyphenol, such as polarity and solubility, so the composition of the extracting solution was optimized. Mirón et al.^17^ found that a 50% methanol content was high enough for an efficient extraction for total polyphenol content determination.

In contrast with the results presented by Mirón et al., previous works devoted to the separation and determination of individual polyphenolic compounds reported that a minimum of 80% methanol content in the extracting solution was necessary to achieve a complete phenolic extraction.^23^ However, as the liquid–liquid interface area for DLLPAE is higher than when a conventional procedure is applied such as liquid–liquid extraction, the total amount of methanol in the extracting solution could be reduced.^17^ To achieve the maximum extraction efficiency for each phenolic compound, the composition of the extracting solution was evaluated, considering three different methanol concentrations: 30, 50 and 75% (w/w). Five replicates were performed for each extracting solution, and the methanolic extracts were then analyzed by HPLC-DAD (Section 2.5). The extracted mg kg^–1^ for each compound when using 30, 50, and 75% methanol in the extracting solutions are summarized in Table 2. As shown, when the methanol content was 30%, the extraction yield reached for ferulic acid; oleuropein, and vanillin decreased when compared with the 75% methanol solution. However, no significant differences were found for 50 and 75% methanol content extracting solution for the same compounds. So, 50:50 methanol/water extracting solution was selected. As it was observed for total phenolic content determination, and in contrast with the observation made by Angerosa et al.,^24^ it was not necessary to increase the percentage of methanol to reach a complete extraction. These discrepancies could be assigned to the fact that, with the DLLAPE, the total interface area was higher than for conventional liquid–liquid extraction methods and, hence, the content of methanol (i.e., the least polar extractant component) could be lowered.

**Table 2:** Amount of Extracted Compounds (mg kg^–1^) by Using 30, 50, and 75% Methanol in the Extracting Solution^a^

	HYR	TYR	oleuropein	vanillic acid	*p*-coumaric Acid	ferulic acid	vanillin
30%	28 ± 5^a^	50 ± 4^a^	0.6 ± 0.1^a^	0.43 ± 0.08^a^	0.18 ± 0.03^a^	0.12 ± 0.02^a^	0.37 ± 0.03^a^
50%	29.3 ± 0.8^a^	53.5 ± 0.9^a^	0.82 ± 0.02^b^	0.46 ± 0.03^a^	0.20 ± 0.01^a^	0.15 ± 0.01^a.b^	0.50 ± 0.02^b^
75%	31 ± 3^a^	55 ± 5^a^	0.89 ± 0.08^b^	0.43 ± 0.08^a^	0.22 ± 0.01^a^	0.16 ± 0.02^b^	0.53 ± 0.08^b^

a Numbers in the same column followed by different letters are statistically different *p* ≤ 0.05 (Tukey’s test).

### 3.2 UHPLC-MS/MS Method Validation Parameters

Table S2 summarizes the main method validation parameters obtained. Linearity was evaluated for each minor polyphenolic compound in the concentration range found in the samples. The correlation coefficients were within the 0.9932–0.9997 range. Intraday and interday reproducibility values were calculated, in terms of relative standard deviation (RSD), for a 25 μg kg^–1^ quality control sample. The intraday and interday RSD values were within the range of 0.93–2.17 and 0.99–2.83%, respectively. One of the advantages of the UHPLC-MS/MS method is the low LOD and LOQ achieved. LOD and LOQ were within the range of 0.0006–0.0244 and 0.0019–0.0814 μg kg^–1^, respectively.

### 3.3 Analysis of Raw EVOO Samples

Samples of raw EVOO were analyzed through the spectrophotometric method making use of Folin-Ciocalteu reagent to determine the TPC values, and the obtained results are summarized in Table 3.

**Table 3:** TPC Values (Expressed as mg Gallic Acid Equivalent kg^–1^ Oil ± Standard Deviation) and Phenolic Substance Concentrations (mg kg^–1^ Oil ± Standard Deviation) in Raw EVOO Samples^a^

oil code	*C*_GAE_	HYR	TYR	oleuropein	vanillic acid	*p*-coumaric acid	ferulic acid	vanillin
A1	95 ± 3^bc^	8.4 ± 0.3^c^	15.2 ± 1.6^bc^	0.48 ± 0.04^b^	0.33 ± 0.02^e^	0.133 ± 0.006^b^	0.0492 ± 0.0005^c^	0.30 ± 0.03^bc^
A2	68.8 ± 0.7^ab^	3.7 ± 0.5^b^	16 ± 3^bc^	0.32 ± 0.03^a^	0.092 ± 0.003^a^	0.064 ± 0.004^a^	0.031 ± 0.003^a^	0.229 ± 0.007^a^
B1	105 ± 7^c^	8.2 ± 0.4^c^	17.6 ± 1.3^cd^	0.60 ± 0.06^c^	0.239 ± 0.010^bc^	0.125 ± 0.007^b^	0.042 ± 0.004^b^	0.315 ± 0.005^cd^
B2	257 ± 5^f^	13.1 ± 1.5^e^	31 ± 2^f^	0.720 ± 0.018^efg^	0.40 ± 0.03^fgh^	0.197 ± 0.015^fg^	0.078 ± 0.007^e^	0.33 ± 0.01^cde^
B3	322 ± 12^g^	36 ± 3^i^	55 ± 4^i^	0.83 ± 0.03^i^	0.407 ± 0.019^hi^	0.18 ± 0.03^e^	0.12 ± 0.02^h^	0.47 ± 0.02^g^
B4	320 ± 30^g^	28.9 ± 1.8^h^	49 ± 2^h^	0.74 ± 0.03^gh^	0.420 ± 0.008^hi^	0.197 ± 0.014^fg^	0.1172 ± 0.0011^h^	0.410 ± 0.002^f^
CR1	180 ± 5^e^	7.8 ± 0.3^c^	14.2 ± 1.4^bc^	0.667 ± 0.017^cd^	0.233 ± 0.010^b^	0.126 ± 0.003^b^	0.0510 ± 0.0004^c^	0.272 ± 0.015^b^
G1	443 ± 20^h^	21 ± 3^f^	124 ± 10^j^	1.21 ± 0.10^j^	0.433 ± 0.019^i^	0.227 ± 0.013^h^	0.137 ± 0.011^i^	0.622 ± 0.013^i^
M1	328 ± 7^g^	8.9 ± 0.5^c^	9.0 ± 0.2^a^	0.65 ± 0.07^cd^	0.26 ± 0.02^cd^	0.150 ± 0.007^c^	0.0481 ± 0.0014^bc^	0.487 ± 0.035^g^
M2	493 ± 20^h^	22.4 ± 1.0^g^	40.2 ± 1.8^g^	0.746 ± 0.011^gh^	0.371 ± 0.009^f^	0.185 ± 0.005^ef^	0.0733 ± 0.0011^de^	0.218 ± 0.008^a^
Mo1	170 ± 7^e^	9.0 ± 1.1^c^	20 ± 2^d^	0.698 ± 0.019^efg^	0.272 ± 0.018^d^	0.168 ± 0.005^d^	0.073 ± 0.008^d^	0.276 ± 0.009^b^
P1	280 ± 10^f^	14.2 ± 0.7^e^	39 ± 3^g^	0.77 ± 0.02^hi^	0.467 ± 0.009^j^	0.21 ± 0.02^h^	0.1005 ± 0.0018^g^	0.353 ± 0.005^e^
P2	280 ± 20^f^	22.8 ± 1.0^g^	31.1 ± 0.8^f^	0.709 ± 0.009^fgh^	0.42 ± 0.03^hi^	0.205 ± 0.008^g^	0.0929 ± 0.0007^f^	0.344 ± 0.002^de^
V1	154 ± 8^de^	12.9 ± 0.9^e^	27.7 ± 1.3^e^	0.61 ± 0.02^cd^	0.39 ± 0.03^fgh^	0.19 ± 0.05f^g^	0.072 ± 0.004^e^	0.28 ± 0.02^b^
V2	315 ± 8^g^	29.1 ± 1.6^h^	54.5 ± 1.3^i^	0.81 ± 0.03^i^	0.41 ± 0.04^hi^	0.197 ± 0.0014^fg^	0.142 ± 0.012^i^	0.46 ± 0.02^g^
GA1	64 ± 5^a^	1.97 ± 0.10^a^	16.4 ± 1.8^bc^	0.37 ± 0.02^a^	0.084 ± 0.005^a^	0.062 ± 0.004^a^	0.043 ± 0.004^b^	0.22 ± 0.03^a^
C1	137.7 ± 1.9^c^	10.2 ± 1.1^d^	13.6 ± 1.5^b^	0.67 ± 0.02^cd^	0.232 ± 0.013^b^	0.155 ± 0.006^c^	0.0506 ± 0.0019^c^	0.56 ± 0.03^h^

a Different letters for a given compound indicate significant differences at *p* < 0.05.

As can be observed, the obtained TPC values ranged from 64 to 493 mg gallic acid equivalent kg^–1^ oil, corresponding to GA1 and M2, respectively. All Alfafara and the mixture containing Grossal and Arbequina presented lower contents, ranging from 64 to 95 mg gallic acid equivalent kg^–1^ oil. These results are in agreement with previous reports in which the corresponding total polyphenols mean values for the analyzed monovarietal Alfafara and Arbequina oils were lower than 250 mg kg^−1^.^17^ However, other authors have reported values of total phenolic content exceeding 400 mg kg^–1^, with Alfafara, Blanquiroja, and Blanqueta Reina being the cultivars with the highest concentration of polyphenols. These results reveal that quite different values of total phenolic content in EVOO samples can be found in the literature depending on several factors such as area and year of cultivation and oil extraction method, among others.^25^ This fact is corroborated in the present work where samples from the same cultivar but from different years and locations showed significant differences in the average values for TPC.

**Table 4:** Reaction Rate Constants (k) and Regression Factor (*R*^2^) for Each Individual Phenolic Compound

	HYR	TYR	oleuropein	vanillic acid	*p*-coumaric acid	ferulic acid	vanillin
*k* (min^–1^)	0.070 ± 0.007d	0.036 ± 0.005a	0.081 ± 0.004e	0.044 ± 0.005b	0.036 ± 0.004a	0.052 ± 0.006c	0.049 ± 0.004bc
*R*^2^	0.994 ± 0.004	0.990 ± 0.005	0.988 ± 0.011	0.988 ± 0.012	0.994 ± 0.004	0.909 ± 0.036	0.796 ± 0.060

Concerning the analyzed samples of Manzanilla and Genovesa cultivars, values of TPC higher than 250 mg kg^–1^ were obtained. The influence of the cultivation year was observed in Manzanilla samples since significantly different values of total phenolic content were obtained for samples from the years 2018 and 2021. The same trend is shown in the studied Blanqueta samples, since statistically different mean values of the total phenolic content of 105, 257, and 320 mg kg^–1^ were obtained for samples cultivated in 2019, 2020, and 2021, respectively. However, no statistically significant differences were detected in the total phenolic content in the Blanqueta samples cultivated in 2021 related to the organic or non-organic production.

Since the Folin-Ciocalteau is not a selective method and not able to discriminate between polyphenols and other possible interferents found in food matrices, the content of some important polyphenols in olive oils was determined to observe the influence of the preservation time.^4,26,27^ It is important to highlight that the present work is mostly focused on minor phenolic compounds and on the study of their degradation behavior when submitted to a frying treatment, since they are those in which a more important lack of information has been detected. For this reason, HYR, TYR, oleuropein, *p*-coumaric acid, ferulic acid, vanillic acid, and vanillin (mg kg^–1^) were quantified by using UHPLC-MS/MS analysis, and the obtained results are detailed in Table 3.

Among the quantified compounds, TYR presented the highest concentration values in all studied raw EVOO samples. The TYR content ranged from 9 to 124 mg kg^–1^ followed by HYR (1.97–36 mg kg^–1^), and lower amounts of oleuropein, vanillic acid, vanillin, *p*-coumaric, and ferulic acid were observed. These results agreed with other works in which TYR and HYR and their derivatives^4^ have been reported as the predominant polyphenols in raw EVOO samples.^25^ In this line, Lozano-Castellón et al.^4^ reported a mean value of 15.2 ± 0.7 mg kg^–1^ for HYR in raw EVOO, and Carrasco-Pancorbo et al.^28^ also confirmed this trend reporting mean values of 17 and 9.8 mg TYR and HYR kg^–1^ raw EVOO, respectively. These data were within the range of values shown in the present work. The highest TYR content (124 ± 10 mg kg^–1^) was observed in the Genovesa sample, also reporting a high mean value of total phenolic content of 443 ± 20 mg gallic acid equivalent kg^–1^ oil.

Concerning the year of harvesting and the time that EVOO samples were kept in the dark at ambient temperature inside their bottles, samples corresponding to three different years were taken. Usually, EVOO is consumed within the first 18 months after its production. However, in local areas, oil samples are stored for longer periods of time, and there are not conclusive quantitative results on the polyphenols degradation under these circumstances. Significant differences were obtained in TYR content among Blanqueta samples from 2019, 2020, and 2021 and between Manzanilla and Villalonga raw EVOO samples from 2019 and 2021, confirming the impact of storage time on the phenolic profile of VOO. This parameter can influence the total polyphenol content but also individual polyphenols content as can be observed in Table 3. Considering the year of cultivation independently of the olive cultivar, significant differences in the mean values of TYR, HYR, vanillin, and ferulic acid content were obtained for samples of year 2021 compared to samples from years 2019 and 2020. Even though changes in phenolic content during storage may depend on other antioxidants present in the sample and on the fatty acid profile, the decreasing content tendency in the mean single polyphenols such as in TYR and HYR as the preservation time increases has been observed in the same way as in other studies. Daskalaki et al.^11^ considered that the air content in the top of the bottle can be enough for continuous degradation of antioxidant compounds during their storage, being more evident for long storage periods.

Although this work is not focused on major compounds, concentration values (mg kg^–1^) of oleacein, oleocanthal, oleuropein aglycone, ligstroside, luteolin, and apigenin are shown in Table S14 for raw samples A2, CR1, M1, M2, and V1, as some examples.

In relation to the quantification of oleocanthal and oleacein, their interaction with water or other polar solvents such as methanol may promote the formation of hemiacetal or acetal derivatives. However, in a previous work carried out by Sánchez de Medina et al.,^29^ the use of methanol–water solutions for polyphenols extraction did not promote the formation of acetals and hemiacetals. Taking it into consideration, by using an extraction method based on methanol–water and further UHPLC separation of polyphenols with a mobile phase based on acetonitrile, the formation of methyl hemiacetal of oleacein, dimethyl acetal of oleacein, methyl hemiacetal of oleocanthal, and dimethyl acetal of oleocanthal was not expected.

The highest concentration values were obtained for oleuropein aglycone (9–32 mg kg^–1^) and oleacein (11–20 mg kg^–1^), followed by luteolin (2–7.2 mg kg^–1^), oleocanthal (1–4 mg kg^–1^), ligstroside (0.3–0.7 mg kg^–1^), and apigenin (0.1–0.4 mg kg^–1^). The highest concentration of oleuropein aglycone was found in sample V1 (31.7 mg kg^–1^), whereas for the compound oleacein, samples A2 and CR1 showed the highest values (19.9 and 19.5 mg kg^–1^, respectively). The obtained results agree with the previous works that have reported the cultivar Alfafara (sample A2) as being one in which oleacein has been found at high levels along with the cultivars Pendolino, Blanqueta, Arbequina, Cerezuela, Kalamon, Caballo, and Koroneiki.^25^ In relation to the amount of luteolin and apigenin present in the studied raw samples, the same trend was observed for these compounds in Picual, Arbequina, Cornicabra, and Hojiblanca oil samples in which these compounds were present in lower amounts in comparison with the values reported for oleuropein aglycone and oleacein.^12^

### 3.4 Evolution of Total Polyphenolic Content with Thermal Treatment

**Figure 1:**
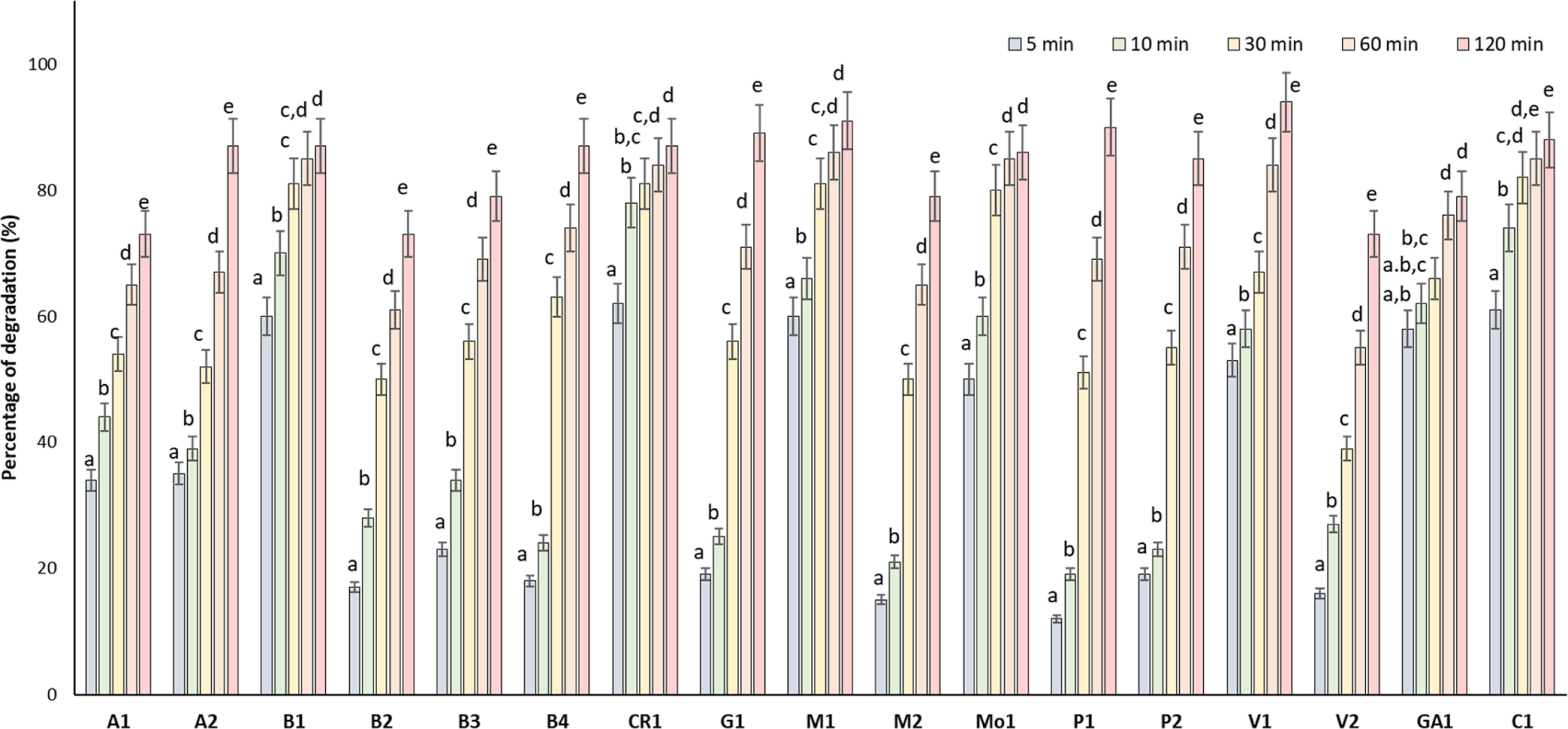
Percentage of degradation of EVOO samples after heat treatment at different degradation times. Different letters for the same degradation time indicate significant statistical differences (*p* < 0.05)

The results obtained after carrying out the home-frying treatment were compared with the content of total polyphenols in the raw EVOO samples. Figure 1 shows the percentages of degradation obtained at different home-frying treatment times for each sample. The relative standard deviation in all cases was lower than 10%.

The total phenolic content analysis of the EVOO, heated at 180 °C, revealed a decrease in phenolic compounds with the frying time. The thermal degradation was produced differently depending on the EVOO sample, without cultivar correlation. For example, after 5 min of thermal treatment, the Alfafara cultivar (A1 and A2) showed 35% degradation for both samples (Figure 1). Moreover, the total content of phenolic compounds decreased significantly within the first 5 min, and a progressive less significant drop in the polyphenolic content was observed for the longest degradation times. For Blanqueta samples (Table 1), the percentage of degradation measured for B2 and B3 was around 20% within the first 5 min; however, for B1 the percentage of degradation was 60% at the mentioned degradation time.

In general, all samples showed statistically significant differences with respect to the non-degraded sample after 5 min of thermal degradation (Figure 1). The Changlot Real (CR1) sample showed the lowest resistance to thermal degradation since, after 5 min of cooking, the percentage of degradation took a value of 63%.

Lozano-Castellón et al.^4^ reported a 75% decrease in TPC values when Hojiblanca EVOO samples were exposed to a temperature of 170 °C for 30 min. As Figure 1 shows, the TPC values for the V2 sample suffered from a 39% decrease, whereas the maximum degradation rate (82%) was observed for the C1 sample, for the same period (30 min). In both studies, a home-frying process has been simulated, thus the exposure to oxygen and light was not controlled. When a deep-fat fryer was used, where the sample exposure to oxygen and light was lower, a 50% decrease in the phenolic content was reported after 3 h of heating treatment.^9^Figure 1 shows the results obtained after 2 h of thermal degradation, indicating that the frying process caused a decrease in total phenolic content higher than 73% for all studied samples.

### 3.5 Changes in the Content of Individual Polyphenols in EVOO Samples with Thermal Treatment

The content of the studied EVOO polyphenols, heated at 180 °C, revealed a decreasing trend over time. A correlation between the varietal composition of the EVOO and the rate of degradation of the different individual minor polyphenols was not found (Tables S4–S10). To evaluate the differences in terms of thermal degradation of phenolic compounds, the mean percentage degradation value was calculated for each minor polyphenol at different thermal degradation times. Variability among samples was expressed as standard deviation. As shown, the percentage of degradation depended on the compound evaluated.

**Figure 2:**
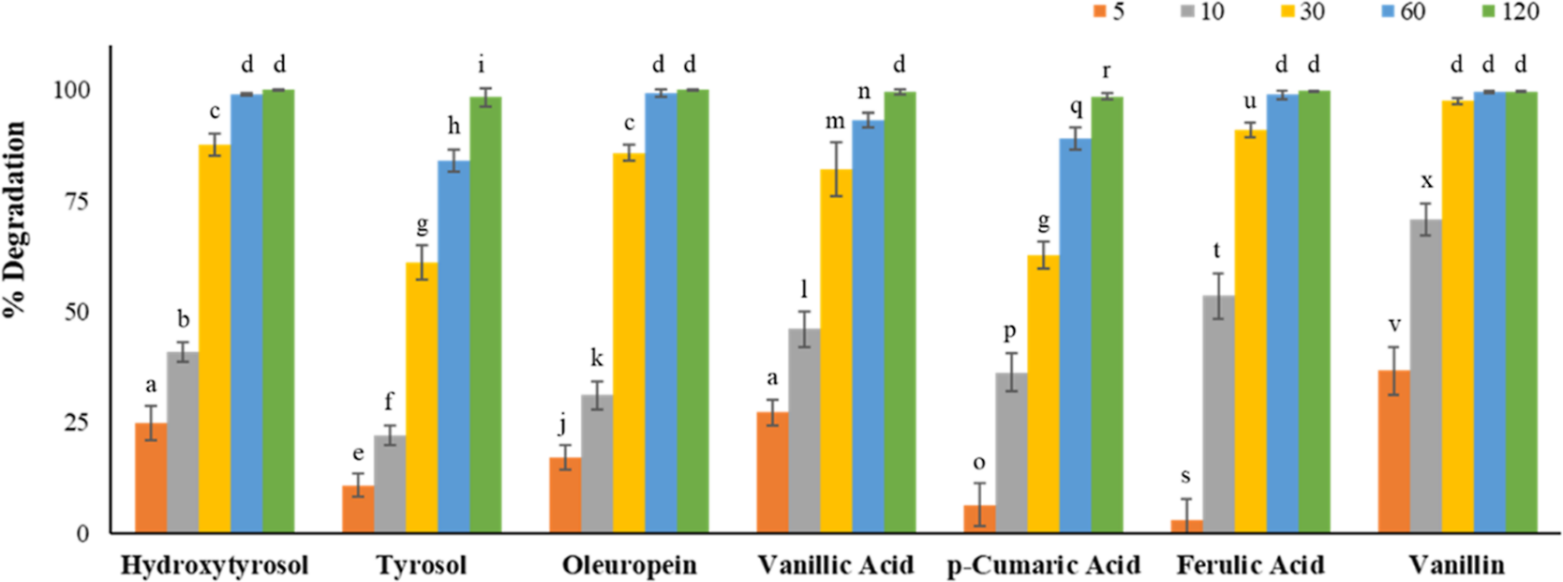
Degradation (%) for the evaluated phenolic compounds. Different letters for the same compound indicate significant statistical differences (*p* < 0.05). Different letters for the same time indicate significant statistical differences (*p* < 0.05).

As Figure 2 and Table S4 show, HYR content rapidly decreased with degradation time. After 5 min of thermal treatment, the content of HYR suffered a 25 ± 4% decrease. A degradation treatment of 10 min was enough to reduce the content of this compound by a 41 ± 2% factor. Gómez-Alonso et al.^30^ reported that the concentration of HYR and its secoiridoides derivatives decreased up to 50–60% after frying at 180 °C for 10 min, whereas 60% of degradation was found by Lozano-Castellón et al.^4^ after 30 min of domestic sautéing at 170 °C. The reduction in the concentration of HYR after 30 min of degradation was higher in the case of the analyzed samples. HYR content was reduced by an 88 ± 2% factor. This fast reduction could be assigned to thermal or oxidative degradation because of its contribution to EVOO stability. Species that do not participate in the stability of the oil could be expected to present a very attenuated degradation curve. This is the case of TYR; however, this compound presented low thermal stability (Figure 2, Table S5). A degradation time of 10 min was sufficient to reduce the concentration of this compound by 11 ± 3%, while to observe a 90% reduction, it was necessary to apply a degradation time longer than 60 min. For this polyphenolic compound, the degradation occurred more gradually than in the case of HYR. These differences have been previously described in the literature.^10,11,28^

Another of the main polyphenols present in olive oil is oleuropein. Thermal degradation of oleuropein was higher than 10% for all of the studied samples after 5 min at 180 °C (Figure 2 and Table S6), and after 60 min of heating, its concentration was reduced by about 99.3 ± 0.8%. These results were like those found by Attya et al.^31^ that reported a reduction in the oleuropein content of 93% after 1 h at 230 °C, although the used temperature was higher than the one applied in this work.

Concerning the other minor polyphenolic compounds, it should be noted that vanillic acid (Figure 2, Table S7) presented a behavior like that of oleuropein. Besides, 5 min at 180 °C were not enough to decrease the content of ferulic acid more than 10% for all of the studied samples. In relation to *p*-coumaric acid (Figure 2, Table S8), the same behavior was observed except for samples A1, B1, and B3. However, 10 min was enough to reduce the concentration of both polyphenols by 36 ± 4% and 54 ± 5%, respectively. To achieve 90% of phenolic content degradation, it was necessary to apply 60 min of thermal treatment to the *p*-coumaric acid, while ferulic acid only required 30 min. Vanillin presented the poorest thermal stability (Figure 2, Table S10). The concentration of this compound was halved after 5 min of thermal treatment. After 30 min, this compound reached degradation ratios of about 100%.

Although major compounds are not the main aim of this work, the concentration (mg kg^–1^) values of some major compounds such as oleacein, luteolin, oleuropein aglycone, oleocanthal, apigenin, and ligstroside in samples A2, CR1, M1, M2, and V1 subjected to frying at 0, 5, 10, 30, 60, and 120 min are shown in Figure S1, as some examples. A significant and progressive concentration decay was found for oleuropein aglycone, oleacein, ligstroside, and luteolin in the mentioned EVOO samples, this decrease being particularly severe after 10 min frying, as reported by other authors.^12^ Manzanilla EVOO (M2) led to the highest concentration decrease for oleuropein aglycone (∼88%) after 10 min, followed by samples A2, CR1, M1 (∼65 to 70%), and V1 (∼50%). In addition, the obtained results pointed out that the thermal treatment influenced particularly the concentration of oleocanthal. This compound showed the same pattern reported in other studies, observing an initial concentration increase and then a lowering tendency until the end of the frying process. These variations could be attributed to the conversion of the pair oleomissional/oleokoronal to oleacein/oleocanthal based on a simple hydrolysis that would be enhanced by frying conditions in terms of temperature and humidity.^10^

### 3.6 Kinetic Studies to Monitor the Degradation of EVOO Samples under Heat Treatment in Terms of Polyphenolic Compound Concentration

By plotting the natural logarithm of the concentration versus heat treatment time, it is possible to obtain the rate constant. (Table 4) reports the mean ± SD of the reaction constant and regression factor obtained for each minor polyphenol. Data for each sample are shown in Tables S11 and S12.

The first-order kinetic degradation model was properly fitted for most of the minor phenolic compounds analyzed in this work. The regression factor values were higher than 0.9 for all phenolic compounds except for vanillin (0.80 ± 0.06). Statistical analysis of the data using ANOVA showed that, since the *p*-value (0.0011) of the *F*-test is lower than 0.05, there was a statistically significant difference in terms of mean reaction constant values obtained for each individual phenolic compound. To determine which means were significantly different from which others, a Fisher’s least significant difference procedure was applied. Two pairs of statistically equivalent reaction constant values were found: *p*-coumaric acid and TYR and ferulic acid and vanillin. Therefore, a similar kinetic degradation behavior was found for these compounds.

EVOO samples from the same cultivar but from different years and locations showed significant differences in the average values for total phenolic content. However, no statistically significant differences were detected in TPC values in samples from the same cultivar and year, considering the organic or non-organic production. In relation to the TYR, HYR, vanillin, and ferulic acid content, no statistically significant differences were obtained in their mean values for samples of the year 2021 in comparison with those from the other studied years, 2020 and 2019, considering the year of cultivation independently of the olive cultivar.

The domestic-frying process reduced the TPC, obtaining a mean reduction value of 94% after frying at 180 °C for 2 h. During the thermal degradation, the content of individual phenolic compounds was reduced. The varietal composition of the EVOOs had no significantly different impact on the rate of degradation of the different individual polyphenols. However, the degradation rate affects different phenolic compounds differently. Kinetic studies showed that the first-order model properly described the degradation of the individual minor compounds. Moreover, the pairs *p*-coumaric acid/TYR and ferulic acid/vanillin presented statistically equivalent reaction constant values.

## Supporting Information Available

Compound name, precursor, and product ions (*m*/*z*) and collision energy (*V*) values; UHPLC-MS method validation parameters; mean values and standard deviation of the individual polyphenols obtained for the samples corresponding to the same harvesting year and independently of cultivar type; concentration (mg kg^–1^) of oleacin, luteolin, oleuropein aglycone, oleocanthal, apigenin, and ligstroside in samples A2, CR1, M1, M2, and V1 subjected to frying by analysis sampled at 0, 5, 10, 30, 60, and 120 min; percentage of HYR, TYR, oleuropein, vanillic acid, *p*-coumaric acid, ferulic acid, and vanillin degradation in EVOO samples after heat treatment at different degradation times; reaction rate constants (k min^–1^) for each individual phenolic compound present in EVOO samples; regression factor (*R*^2^) for each individual phenolic compound present in EVOO samples; optimum operating conditions for the determination of polyphenols and metals through the DLLAPE method; concentration values (mg kg^–1^ oil ± standard deviation) of major phenolic compounds in raw EVOO samples; and chromatogram obtained by using HPLC-DAD (280 nm) (PDF)
