# Supplementary material for: Application of Dispersive Liquid–Liquid Aerosol Phase Extraction to the Analysis of Total and Individual Phenolic Compounds in Fried Extra Virgin Olive Oils
Source: J Agric Food Chem. 2023 Jul 3;71(28):10742–50. doi: 10.1021/acs.jafc.3c02634 (PMC10360154; doi:10.1021/acs.jafc.3c02634)

Application of the Dispersive Liquid-Liquid Aerosol Phase Extraction to the Analysis of Total and Individual Phenolic Compounds in Fried Extra Virgin Olive Oils

Raquel Sánchez; Ana Beltrán Sanahuja*; María Soledad Prats Moya; José-Luis Todolí

Department of Analytical Chemistry, Nutrition and Food Sciences, P.O. Box 99, 03080, Alicante, Spain

Table S1. Compound name, precursor, and product ions (m/z) and collision energy (V) values.

Compound Name	Precursor Ion (m/z)	Product Ion (m/z)	Collision Energy (V)
Ligstroside	523	361	6
Ligstroside	523	112	14
Oleuropein Aglycone	377	307	4
Oleuropein Aglycone	377	275	4
Ligstroside Aglycone	361	291	16
Ligstroside Aglycone	361	259	2
Oleacein	319	195	0
Oleacein	319	69	20
Oleocanthal	303	165	0
Oleocanthal	303	59	16
Luteolin	285	133	35
Apigenin	269	117	35
Oleuropein	539.17	376.9	16
Oleuropein	539.17	307.2	20
Oleuropein	539.17	275.3	24
Oleuropein	539.17	149	24
Ferulic Acid	193.05	178	12

Ferulic Acid	193.05	149	8
Ferulic Acid	193.05	133.9	16
Vanillic Acid	167.03	152	12
Vanillic Acid	167.03	123.1	8
Vanillic Acid	167.03	91	20
p-Cumaric Acid	163.04	119	16
p-Cumaric Acid	163.04	117	36
p-Cumaric Acid	163.04	92.9	40
p-Cumaric Acid	163.04	91.1	32
HYR	153.05	123	16
HYR	153.05	94.9	20
HYR	153.05	92.9	28
HYR	153.05	76.9	24
Vanillin	151.04	136	12
Vanillin	151.04	108	24
Vanillin	151.04	92.1	24
TYR	137.06	119.1	16
TYR	137.06	107	16

Table S2. UHPLC-MS method validation parameters.

Standard	Linearity (µg kg-1)	R^2	LOD (µg kg-1)	LOQ (µg kg-1)	Reproducibility (RSD %)	
					Intraday	*Interday*
HYR	0.10-115.23[a]<br>5-4991[b]	0.9935[a]<br>0.9994[b]	0.0244	0.0814	1.02	1.13
TYR	0.11-114.23[a]<br>4-5058[b]	0.9959[a]<br>0.9997[b]	0.0046	0.0153	1.57	1.71
Oleuropein	0.10-112.32	0.9992	0.0006	0.0019	1.62	1.89
Vanillic Acid	0.11-104.23	0.9987	0.0203	0.0678	2.17	2.83
p-Coumaric Acid	0.11-105.47	0.9932	0.0213	0.0711	0.93	0.99
Ferulic Acid	0.11-10.43	0.9985	0.0014	0.0046	1.18	1.52
Vanillin	0.10-99.64	0.9991	0.0212	0.0707	0.97	1.63

[a] Calibration curve employed for HYR or TYR concentration determination for the samples with low polyphenol content.

[b] Calibration curve employed for HYR or TYR concentration determination for the samples with high polyphenol content.

Table S3 Mean values and standard deviation of the individual polyphenols obtained for the samples corresponding to the same harvesting year and independently of cultivar type

Polyphenols	Aging	N	Mean* (mg kg^{-1})	Standard deviation	Minimum (mg kg^{-1})	Maximum (mg kg^{-1})
	2019	24	7.8a	3.3	1.9	13.3
HYR	2020	3	13.1a	0.4	12.7	13.4
	2021	21	25.0b	6.9	13.9	37.7
	2019	24	17.7a	4.4	13.1	27.7
TYR	2020	3	31.4a	0.4	30.9	31.7
	2021	21	56.4b	29.5	30.8	128.4
	2019	24	0.55a	0.14	0.31	0.70
Oleuropein	2020	3	0.72ab	0.01	0.72	0.72
	2021	21	0.83b	0.17	0.71	1.26
	2019	24	0.23a	0.11	0.08	0.40
Vanillic acid	2020	3	0.39ab	0.16	0.14	0.16
	2021	21	0.42b	0.03	0.06	0.232
	2019	24	0.12a	0.10	0.06	0.22
Coumaric acid	2020	3	0.19b	0.02	0.20	0.20
	2021	21	0.20b	0.03	0.17	0.23
	2019	24	0.30a	0.10	0.21	0.57
Vainillin	2020	3	0.32a	0.01	0.32	0.33
	2021	21	0.41ab	0.12	0.22	0.63
Ferrulic acid	2019	24	0.051a	0.013	0.03	0.08
	2020	3	0.077a	0.001	0.077	0.08
	2021	21	0.11b	0.02	0.07	0.14º

*Different letters for the same polyphenol indicate significant differences at $p<0.05$.

Figure S1. Concentration (mg kg^{-1}) of oleacin. luteolin. oleuropein aglycone. oleocanthal. apigenin and ligstroside in samples A2. CR1. M1. M2 and V1subjected to frying by analysis sampled at 0, 5, 10, 30, 60 and 120 min.

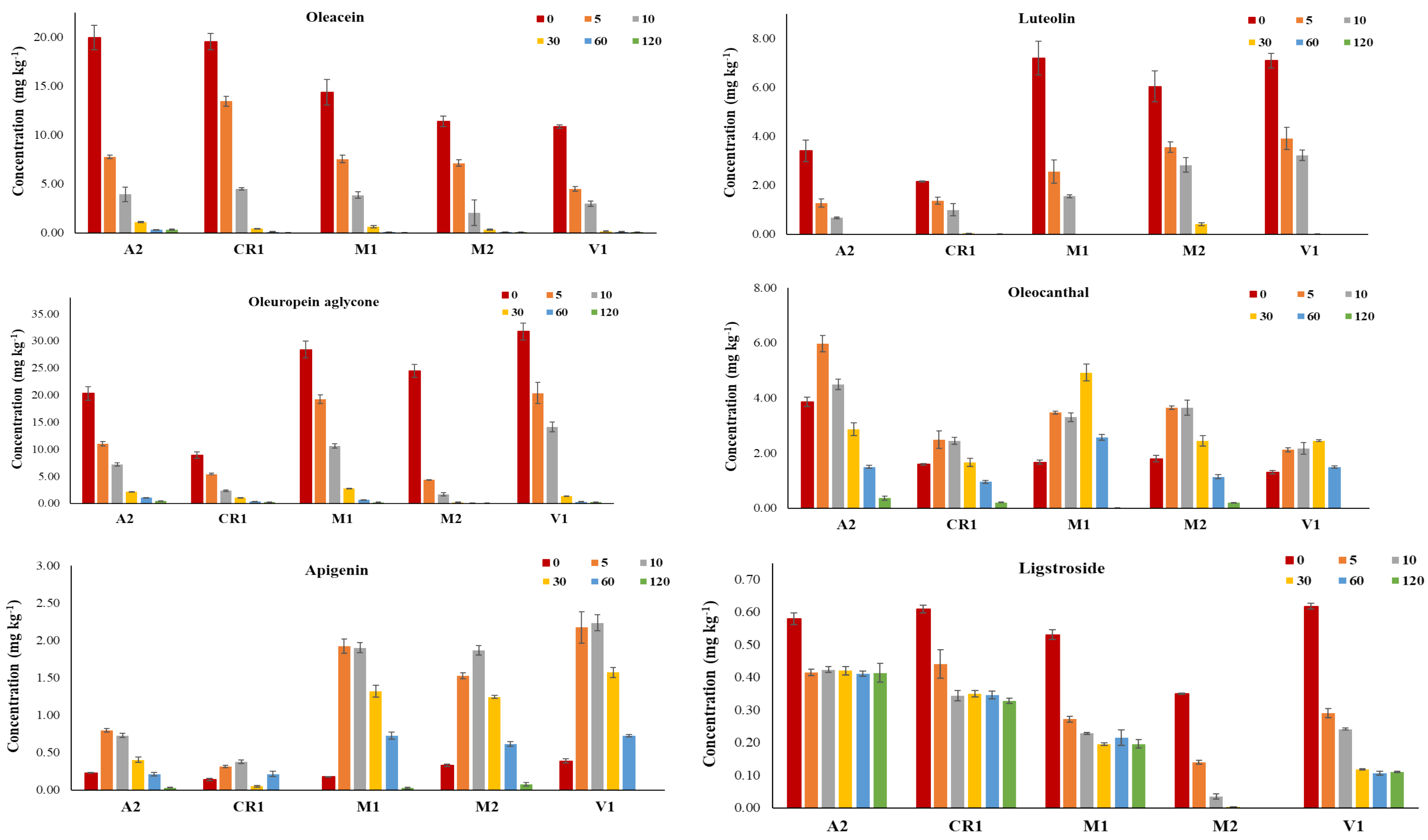

Table S4. Percentage of HYR degradation in EVOO samples after heat treatment at different degradation times.

**Degradation time (min)**	*A1*	*A2*	*B1*	*B2*	*B3*	*B4*	*CR1*	*G1*	*M1*	*M2*	*Mo1*	*P1*	*P2*	*V1*	*V2*	*GA1*	*C1*
5	27.9±0.9	17.9±1.3	27.8±1.4	18.6±0.5	24.6±0.5	26.6±0.5	25±2	21.5±1.3	31.5±1.9	18.1±0.4	22.3±1.2	27.1±1.3	30.2±1.6	24.3±1.0	26.3±0.6	21.4±0.7	25.0±1.1
10	39.9±1.3	41±3	39.6±1.9	38.0±1.1	37.6±1.4	40.0±0.9	42.7±1.7	42±4	46±4	41.1±1.6	42±2	40.4±0.9	39.2±1.6	42.9±1.5	39.9±0.9	39.6±1.0	36.3±1.7
30	88±3	88±6	89±5	90±5	89±3	88.9±1.7	88±3	87±9	90±4	86.0±1.8	80±7	83±3	87±5	90±9	88±2	88.4±1.9	89±9
60	99±3	99±8	99±5	99±6	99±3	98±7	99±4	99±6	99±6	99.0±1.8	99±5	99±6	98±4	99±6	99±2	99±5	99±5
120	100±8	100±8	100±5	100±6	99±4	100±3	100±4	100±7	100±9	100±4	100±5	100±6	100±5	100±9	100±8	99±8	100±10

Table S5. Percentage of TYR degradation in EVOO samples after heat treatment at different degradation times.

**Degradation time (min)**	*A1*	*A2*	*B1*	*B2*	*B3*	*B4*	*CR1*	*G1*	*M1*	*M2*	*Mo1*	*P1*	*P2*	*V1*	*V2*	*GA1*	*C1*
5	5.1±0.5	4.9±0.4	10.8±0.2	9.5±0.2	10.3±0.4	10.6±0.2	16.2±0.7	8.9±0.3	11.2±0.6	10.9±0.2	13.1±0.5	11.2±0.5	9.0±0.2	11.7±0.2	13.3±0.4	12.0±0.5	12.7±0.5
10	20±2	22.2±1.7	25.6±0.8	21.7±0.8	21.4±0.8	22.5±0.2	21.5±1.1	22.7±0.7	20.2±1.7	22.1±0.5	23.3±1.3	22.7±1.2	19.3±0.6	25.0±1.1	20.5±0.5	26.3±1.2	20.1±0.8
30	49±6	61±6	66.4±1.4	66.2±1.8	64±3	62.9±1.0	62±3	61±2	61±2	60±3	58±3	62±3	59±3	63.0±1.7	66±3	61±4	57±6
60	79±8	86±8	83±5	85±3	84±4	86±4	80±6	86±3	85±4	84±6	81±6	83±4	85±5	84.7±1.0	84±2	87±6	86±3
120	91±8	99±8	99±3	99.0±1.4	99±4	98.6±1.6	99±5	99±7	99±4	99±3	99±6	99±5	99±3	99±6	99±3	99±5	99±4

Table S6. Percentage of oleuropein degradation in EVOO samples after heat treatment at different degradation times.

**Degradation time (min)**	*A1*	*A2*	*B1*	*B2*	*B3*	*B4*	*CR1*	*G1*	*M1*	*M2*	*Mo1*	*P1*	*P2*	*V1*	*V2*	*GA1*	*C1*
5	13.6±1.4	12.2±0.4	19.0±0.7	13.93±0.08	18.5±0.5	14.5±0.4	20.5±0.4	20.3±0.5	17.0±0.5	15.9±0.2	17.2±0.2	17.4±0.6	12.4±0.2	19.0±0.3	18.1±0.4	20.3±0.5	19.1±0.5
10	40±4	31.6±1.2	35.2±1.2	28.44±0.15	30.7±0.6	29.7±0.5	28.0±0.4	30.7±0.7	30.4±0.9	33.7±0.7	30.9±0.4	30.5±0.4	29.5±0.3	31.2±0.2	29.7±0.9	31.6±0.6	26.8±0.5
30	90±12	87±3	84±3	85±3	85±5	87±4	84±3	85±3	86±2	86.5±1.2	82.8±1.0	85±6	82.9±0.2	84.7±0.9	85.2±1.8	86±3	85.4±1.7
60	100±10	99±4	99±4	99.3±1.8	99.4±1.8	99±9	99±3	99±4	99±5	99±3	99±4	99±3	99.4±0.4	99±2	99±7	99.6±1.9	96±2
120	100±12	100±15	100±9	100±11	100±10	100±8	100±	100±8	100±13	100±6	100.0±1.9	100.0±1.8	100±11	99±4	100±9	100±9	100±5

Table S7. Percentage of vanillic acid degradation in EVOO samples after heat treatment at different degradation times.

**Degradation time (min)**	*A1*	*A2*	*B1*	*B2*	*B3*	*B4*	*CR1*	*G1*	*M1*	*M2*	*Mo1*	*P1*	*P2*	*V1*	*V2*	*GA1*	*C1*
5	27±2	29.7±0.6	26.9±0.5	23.5±1.0	26.5±1.0	27.3±0.6	25±2	29.8±0.7	27.4±1.8	30.6±0.4	27.6±1.2	24.7±0.6	31.3±0.6	26.3±1.1	34±2	28.7±0.5	26.7±1.2
10	52±4	44.2±0.9	49.4±0.8	51±3	49.4±1.8	47.9±0.3	35.9±1.5	47.4±0.8	46±5	48.2±0.7	48.3±1.5	45.4±0.2	45.6±1.4	40.5±1.5	39±2	45.5±1.5	43.9±1.1
30	84±8	85±3	79.5±1.8	80±4	82±2	83.0±0.7	58.7±1.9	89.6±0.6	85±3	80.9±1.2	85±4	86.0±1.3	85.7±1.9	84±3	84±6	81±3	81.0±1.7
60	91±7	92±3	93±3	94±5	92±2	94±5	93±3	96±4	92±17	91.6±0.7	92±3	94±3	92.3±1.7	97±4	94±8	90.9±1.1	94.1±1.6
120	98±10	99.8±0.5	99±4	99±7	99±7	99.7±1.2	99.6±1.8	100.0±1.7	100±10	99±6	99±6	99±8	99.7±1.4	99±4	100±12	99±3	100±7

Table S8. Percentage of p-coumaric acid degradation in EVOO samples after heat treatment at different degradation times.

**Degradation time (min)**	*A1*	*A2*	*B1*	*B2*	*B3*	*B4*	*CR1*	*G1*	*M1*	*M2*	*Mo1*	*P1*	*P2*	*V1*	*V2*	*GA1*	*C1*
5	19.9±0.9	2.35±0.02	16.7±0.4	6.0±0.2	11.1±0.8	3.333±0.015	4.8±0.2	1.254±0.011	4.8±0.2	8.5±0.2	2.3±0.2	9.6±0.3	6.9±0.2	5.7±0.3	4.3±0.2	3.74±0.14	4.9±0.2
10	43±2	36.5±0.4	32.2±0.7	36.0±0.5	39±4	36.9±0.5	31.9±0.4	30.4±0.9	37±4	37.7±0.4	26.4±0.4	42.3±0.5	41.8±0.4	40±3	38.2±0.5	36.2±1.3	34.8±0.3
30	68±2	59.6±1.5	68±3	66±4	64±5	62±4	59.2±0.9	64.0±1.3	63±4	63.4±1.7	54.7±0.8	62.5±1.9	63.9±0.3	63±4	62±4	62.7±0.8	62±2
60	92±4	89.4±1.3	88±4	90±3	80±5	88±3	90±7	89.9±1.9	90±2	89±2	89±4	90.2±0.5	89.3±0.9	90±	87.0±0.8	90±4	90.1±0.7
120	99±11	98.7±1.0	99. ±3	99±3	97±8	97±3	98±2	99±4	99±10	97.8±1.9	99±3	99±4	98.5±1.2	99±7	98±4	98±5	99±9

Table S9. Percentage of ferulic acid degradation in EVOO samples after heat treatment at different degradation times.

**Degradation time (min)**	*A1*	*A2*	*B1*	*B2*	*B3*	*B4*	*CR1*	*G1*	*M1*	*M2*	*Mo1*	*P1*	*P2*	*V1*	*V2*	*GA1*	*C1*
5	8.05±0.09	7.8±0.3	8.84±0.15	-1.15±0.02	0.85±0.07	3.06±0.03	9.24±0.12	4.0±0.2	6.9±0.2	4.05±0.03	1.66±0.07	-3.8±0.2	-0.645±0.004	2.57±0.02	1.55±0.02	5.0±0.2	1.35±0.03
10	50±2	55±3	50.3±1.2	49.0±0.6	55±4	54.0±1.3	52.9±1.4	55.1±0.9	58±4	52.2±1.8	43±3	48.5±0.4	54.9±0.8	56.8±0.6	56.5±1.4	52±3	62±4
30	88±5	90±2	90.0±1.6	91.0±1.2	87±8	94±8	92.4±1.2	90±3	91±5	91.5±0.8	89±5	91±4	91.6±0.6	91.5±0.9	91.3±1.7	92±4	90.8±1.6
60	95±4	99±3	98.7±1.8	98.9±1.7	99±12	99±5	99.2±1.4	99.1±1.9	99±6	99±3	99±5	99±6	99.0±0.6	99.0±1.8	99±3	99±4	99±6
120	100±11	99±6	100±8	100±8	99±8	99±3	99.7±1.6	99±5	99±5	99±2	100±5	99±11	99±5	99±5	100.0±1.3	100±4	100±6

Table S10. Percentage of vanillin degradation in EVOO samples after heat treatment at different degradation times.

**Degradation time (min)**	*A1*	*A2*	*B1*	*B2*	*B3*	*B4*	*CR1*	*G1*	*M1*	*M2*	*Mo1*	*P1*	*P2*	*V1*	*V2*	*GA1*	*C1*
5	34±3	39.9±1.4	27.2±0.7	43.4±0.7	40.3±0.6	43.1±1.1	40.4±0.3	42±2	37.7±1.8	28.9±0.9	28.1±0.9	32.6±0.2	32.6±0.7	41.3±1.5	33.0±0.6	42±2	42.4±1.1
10	78±8	69.1±1.0	64.4±1.3	67±3	75±4	72.1±0.3	70±2	70.3±0.6	70±3	68.5±1.5	74.1±1.3	71.9±0.4	71.5±0.6	74±2	74.1±1.4	73±3	71±2
30	95±9	97.8±1.3	97.2±1.4	97±3	97±3	97.9±1.1	97.6±1.9	97.8±1.0	98±4	98±3	97±3	97±4	97.7±0.4	98±3	97±6	97±6	98±2
60	98±10	99±9	99±4	99±6	99±6	99.5±0.6	99±4	99.5±1.0	99±7	99±2	99±6	99±6	99±5	99±3	99±3	100±4	100±5
120	100±13	100±3	99±2	99.8±1.4	100±10	99±4	100±3	99±3	99±5	99±3	100±5	100±11	99.7±0.5	100±4	99±3	100±4	100±6

Table S11. Reaction rate constants (k min^{-1}) for each individual phenolic compound present in EVOO samples.

	**HYR**	**TYR**	**Oleuropein**	**Vanillic Acid**	***p*-Coumaric Acid**	**Ferulic Acid**	**Vanillin**
A1	0.073	0.021	0.097	0.031	0.047	0.047	0.043
A2	0.067	0.037	0.080	0.049	0.037	0.050	0.047
B1	0.092	0.035	0.079	0.040	0.038	0.049	0.045
B2	0.079	0.038	0.081	0.041	0.038	0.052	0.051
B3	0.063	0.037	0.077	0.039	0.028	0.052	0.051
B4	0.062	0.036	0.082	0.048	0.030	0.048	0.049
CR1	0.067	0.035	0.080	0.047	0.033	0.051	0.049
M1	0.069	0.040	0.080	0.047	0.038	0.050	0.048
M2	0.069	0.038	0.079	0.043	0.032	0.049	0.052
Mo1	0.067	0.038	0.080	0.048	0.038	0.053	0.049
P1	0.067	0.038	0.082	0.046	0.036	0.049	0.050
P2	0.067	0.039	0.079	0.047	0.035	0.054	0.050
V1	0.072	0.038	0.081	0.050	0.037	0.047	0.047
V2	0.068	0.039	0.084	0.048	0.033	0.077	0.039
GA1	0.064	0.040	0.082	0.035	0.034	0.052	0.053
C1	0.072	0.040	0.077	0.049	0.042	0.054	0.042

Table S12. Regression factor (R^2) for each individual phenolic compound present in EVOO samples.

	**HYR**	**TYR**	**Oleuropein**	**Vanillic Acid**	***p*-Coumaric Acid**	**Ferulic Acid**	**Vanillin**
A1	0.9906	0.9774	0.9466	0.9434	0.9946	0.9808	0.8795
A2	0.9908	0.9960	0.9862	0.9865	0.9957	0.9075	0.7859
B1	0.9900	0.9929	0.9885	0.9946	0.9993	0.9208	0.7597
B2	0.9990	0.9920	0.9948	0.9907	0.9986	0.9227	0.8839
B3	0.9838	0.9930	0.9911	0.9897	0.9907	0.9214	0.8523
B4	0.9953	0.9981	0.9954	0.9961	0.9869	0.8532	0.7843
CR1	0.9948	0.9835	0.9922	0.9900	0.9886	0.8896	0.7924
M1	0.9939	0.9901	0.9930	0.9877	0.9972	0.9193	0.7750
M2	0.9940	0.9899	0.9975	0.9929	0.9921	0.8878	0.8168
Mo1	0.9916	0.9782	0.9897	0.9861	0.9950	0.9090	0.8244
P1	0.9942	0.9864	0.9952	0.9902	0.9948	0.8843	0.7983
P2	0.9990	0.9918	0.9883	0.9859	0.9954	0.9315	0.8083
V1	0.9967	0.9924	0.9865	0.9914	0.9960	0.8685	0.8027
V2	0.9970	0.9884	0.9847	0.9943	0.9964	0.9928	0.6816
GA1	0.9854	0.9924	0.9842	0.9853	0.9938	0.8516	0.8460
C1	0.9987	0.9903	0.9789	0.9980	0.9960	0.9247	0.6458

Table S13. Optimum operating conditions for the determination of polyphenols and metals through the DLLAPE method. Taken from the reference 12.

Variable	Optimal value
Extraction time/ s	90
Methanol concentration in the extracting solution/ %methanol:water (w/w)	50:50
Mass of extracting solution/ g	0.7
Hexane added/ mL	1.0
Nebulizer tip e sample level gap. d/ mm	15
Nebulizer liquid flow rate/ mL min^{-1}	0.9
Nebulizer gas flow rate/ L min^{-1}	0.3

Table S14. Concentration values (mg kg^{-1} oil ± standard deviation) of major phenolic compounds in raw EVOO samples.

**Oil Code**	**Oleacein /mg kg^{-1}**	**Luteolin /mg kg^{-1}**	**Oleocanthal /mg kg^{-1}**	**Oleuropein aglycone /mg kg^{-1}**	**Apigenin /mg kg^{-1}**	**Ligstroside /mg kg^{-1}**
A2	19.9 ± 1.2c	3.4 ± 0.4b	3.85 ± 0.17c	20.3 ± 1.3b	0.228 ± 0.006c	0.578 ± 0.018c
CR1	19.5 ± 0.8c	2.15 ± 0.02a	1.59 ± 0.04b	8.9 ± 0.6a	0.140 ± 0.016a	0.609 ± 0.012d
M1	14.3 ± 1.3b	7.2 ± 0.7c	1.66 ± 0.08b	28.4 ± 1.5d	0.178 ± 0.004b	0.531 ± 0.016b
M2	11.4 ± 0.5a	6.0 ± 0.6c	1.79 ± 0.11b	24.4 ± 1.2c	0.331 ± 0.018d	0.350 ± 0.003a
V1	10.82 ± 0.18a	7.1 ± 0.3c	1.30 ± 0.07a	31.7 ± 1.5e	0.39 ± 0.03e	0.618 ± 0.010e

*Different letters for a given compound indicate significant differences at $p<0.5$

Figure S2. Chromatogram obtained by using HPLC-DAD (280 nm). 1: HYR; 2:TYR; 3: Syringic Acid; 4: Oleurepein dicarboxymethylated aglycone (DAFOA) ; 5: Oxidized DAFOA; 6: Dialdehyde form of ligstroside aglycone (DAFLA); 7:Ligstroside aglycone; 8,9,10: Oleuropein aglycone; 11: Ligstroside aglycone isomer.

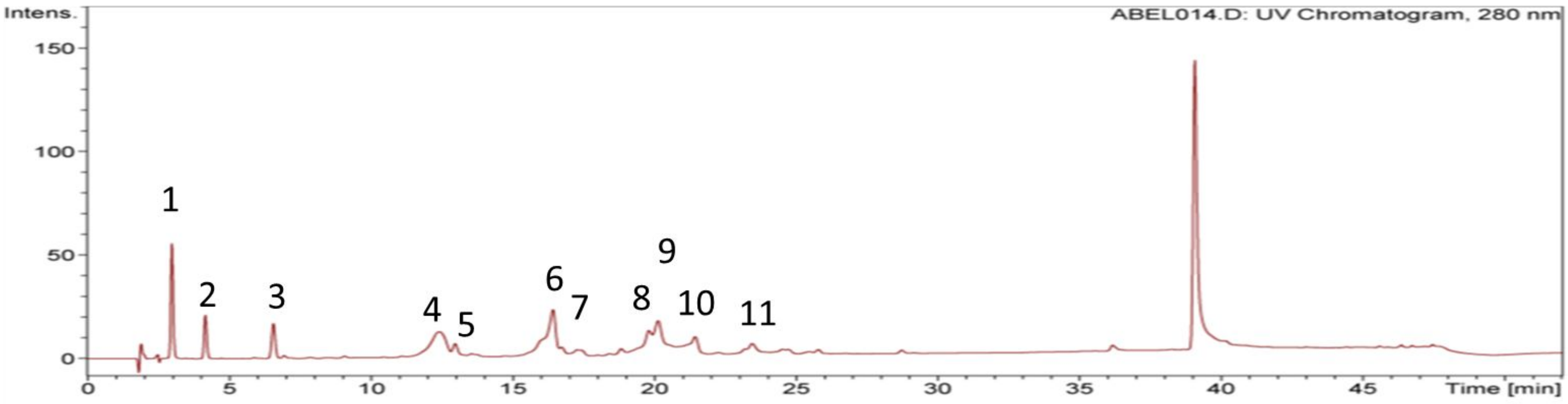

Supplement: Supplementary file 1 — jf3c02634_si_001.pdf [file jf3c02634_si_001.pdf]
